# Xylogenesis of *Pinus koraiensis* is delayed with elevation increase but advanced in smaller rather than larger age

**DOI:** 10.3389/fpls.2026.1816351

**Published:** 2026-05-19

**Authors:** Xiangyi Li, Kexin Jin, Yuxin Bai, Guanhua Dai, Caiyi Xu, Kunyu Pang, Hongqi Sun, Min Xu, Xiaochun Wang

**Affiliations:** 1 Key Laboratory of Sustainable Forest Ecosystem Management (Ministry of Education), College of Ecology, Northeast Forestry University, Harbin, China; 2 Institute of Applied Ecology, Chinese Academy of Sciences, Shenyang, China; 3 Changbai Mountains Nature Conservation Management Center, Shenyang, China

**Keywords:** cambium, phenology, tracheid diameter, tracheid wall thickness, xylem

## Abstract

**Introduction:**

Tree cambial activity and xylem development are regulated by both external environment and internal factors, though their synergistic mechanisms remain unclear.

**Methods:**

This study was conducted during the 2024 growing season across three altitudinal gradients (750 m, 950 m, 1150 m) in Changbai Mountain. We selected Korean pine (Pinus koraiensis) individuals of different ages, sampled at intervals of one to two weeks, and monitored the dynamics of cambial activity and xylem formation processes.

**Results:**

The results showed that with increasing elevation, the effect of decreasing temperature on xylem growth of Korean pine was significant. For every 200 m increase in elevation, the spring onset of cambial activity in Korean pine was delayed by approximately 13 days, while the cessation of xylem growth in autumn advanced by 8-12 days. These changes shortened the growing season length by 12-19 days and further reduced the radial xylem growth of large and small trees by 278 μm and 54 μm, respectively. Compared with low elevation (750 m), the low-temperature environment at high elevation (1150 m) accelerated the earlywood to latewood transition, shortening the transition period by 14 days. Meanwhile, the expansion rates of earlywood and transition area tracheids were affected, decreasing by 1.69 μm/d and 1.63 μm/d, respectively, with corresponding reductions in tracheid diameter of 4.36 μm and 3.5 μm. To adapt to cold stress, Korean pine at high elevation enhanced the mechanical support capacity of cell walls, manifested as increased latewood cell wall thickness. Compared with large trees, small trees exhibited earlier cambial activity onset and later cessation, with a growing season duration 16-27 days longer, thereby producing greater radial xylem growth. Large trees, in contrast, allocated more resources to structural maintenance and stress resistance, as reflected by a 2.7% reduction in the proportion of time spent on earlywood tracheid expansion and a 3.07 μm decrease in tracheid diameter, indicating differences in growth strategies between age classes. Temperature was the key driver of these elevational patterns, and age-related differences in temperature response reflect contrasting adaptive strategies of small-age and large-age Korean pine across elevations.

**Discussion:**

This study elucidates divergent growth responses of Korean pine to climate and aids in predicting broad-leaved Korean pine forest dynamics.

## 1 Introduction

The radial growth of trees is dynamic and highly sensitive, regulated by a combination of environmental factors—such as temperature and moisture—and intrinsic tree characteristics—including species and age (Huang et al., 2020). To date, research on environmental effects on wood formation has focused primarily on phenological traits, such as cambial activity, the timing of xylem cell initiation, cessation, and the overall duration of the growing period (Arnold and Mauseth, 1999; Eckes-Shephard et al., 2022; Buttò et al., 2025). Studying phenology along altitudinal gradients is of great ecological value because air temperature and precipitation vary with elevation and topography (Körner, 2007; Yao et al., 2016). This approach provides an efficient way to reveal the mechanisms underlying tree radial growth responses to future climate warming. For instance, Moser et al. (2010) reported that the onset of the growing season was delayed by 3–4 days per 100 m increase in elevation. Based on the temperature lapse rate recorded in the central Swiss Alps (0.5 °C per 100 m), this corresponds to an approximately 7-day extension of the growing season per 1 °C rise in temperature. Precipitation typically increases with elevation up to a mid-altitude maximum, then decreases toward summits (Barry, 2008). This orographic effect causes air to cool and condense on windward slopes, but moisture depletion reduces rainfall higher up. The peak altitude varies with climate and latitude (Körner, 2007; Yao et al., 2016; Moser et al., 2010). Therefore, investigating how factors like altitude and tree size individually or interactively affect xylem formation is crucial for understanding tree growth responses and forest ecosystem adaptation under climate change.

It is widely recognized that higher altitudes tend to delay the onset of cambial activity in the spring, advance its cessation in the autumn, and thus shorten the total period of cell differentiation and wood formation (Rossi et al., 2008b; Prislan et al., 2018). In high-altitude environments, low temperatures are considered a key factor inhibiting cambial reactivation (Huang et al., 2020), while late-season cold and frost often induce earlier dormancy (Nilsson, 2022). Concurrently, cell enlargement rates decrease significantly at high elevations (Rossi et al., 2008b; Li et al., 2021). The cell wall thickening stage is also compressed due to the shortened growing season and reduced supply of photosynthetic assimilates (Cuny et al., 2014). Some studies further indicate that under extreme high-altitude conditions, latewood cells may exhibit reduced lignification and thinner cell walls, consequently affecting wood density (Ren et al., 2019; Zhang et al., 2021a).

Most existing studies along elevation gradients have focused on trees of similar tree age, with limited systematic investigation into the interactive effects of tree age and climatic gradients on growth—even though tree age has been confirmed as a significant modulator of tree growth (Stephenson et al., 2014; Zeng et al., 2018). Observations of different age classes of Korean pine (*Pinus koraiensis*) revealed that younger pines exhibit longer growing seasons, extended cell differentiation periods, and an earlier start to cambial activity in spring (Zhang et al., 2024). With increasing tree age, the seasonal timing of cambial activity undergoes systematic adjustments, with the most pronounced differences reflected in the onset of cambial reactivation, the duration of the growing season, and the production of xylem cells (Rossi et al., 2008a). Numerous intra-annual radial growth monitoring studies have shown that, compared with older trees, younger individuals generally initiate cambial activity earlier, experience a longer growing season, and produce more xylem cells (Zeng et al., 2018; David et al., 2024). For example, studies on *Pinus tabuliformis* in the Hasi Mountains and *Juniperus przewalskii* in the Qilian Mountains, China both demonstrated that young trees begin cambial activity earlier and exhibit faster growth rates (Zeng et al., 2018). Older Korean pine trees, with their more developed root and canopy structures, hold advantages in resource acquisition. They can adapt to cold or arid conditions by adjusting xylem structural traits (Li Y. et al., 2024; Zhang et al., 2024; Omelko et al., 2025; Malik et al., 2020)—such as increasing tracheid length and optimizing tracheid arrangement—thereby improving hydraulic efficiency and stress resilience (Zhang et al., 2023; Malik et al., 2020).

Korean pine is a key constructive species in temperate mixed coniferous-broadleaf forests in China. It plays an irreplaceable role in maintaining forest ecosystem stability, carbon sequestration, and biodiversity (Wang et al., 2019). Previous studies on environmental effects on wood formation have mostly focused on its phenological characteristics, namely the seasonal onset, ending, and duration of cambial activity and wood formation (Deslauriers et al., 2008; Rossi et al., 2008b, 2016, 2007). These studies have shown that this process exhibits considerable plasticity in response to temperature variation. For example, in cold environments, the growing season is shortened because wood formation begins later and ends earlier. However, our understanding of the mechanisms underlying tree-ring structure formation during xylogenesis remains very limited. Specifically, the duration and rate of cell enlargement determine the final size of xylem cells, whereas the duration and rate of cell-wall thickening determine their final mass. While several studies have examined Korean pine growth along elevation gradients under climate change (Zhou et al., 2018; Yu et al., 2013), comprehensive observations and comparisons of cambial activity, earlywood/latewood transition, cell structural traits and lignification processes across different tree age of Korean pine along such gradients are still lacking.

Based on cellular observations of cambial phenology and xylem formation in large- and small-age Korean pines along different elevation gradients in the Changbai Mountains, this study aims to investigate how environmental variation (e.g., temperature and water availability) and endogenous factors (e.g., tree age) jointly influence xylem development in Korean pine. Specific objectives include: (1) to analyze the effects of altitude (reflecting variations in temperature and precipitation) on cambial phenology and successive stages of xylem formation in different-sized Korean pines; and (2) to examine how the rates of cell differentiation (enlargement and wall thickening) and the proportional duration of earlywood, transition, and latewood formation regulate the final xylem cell structure under varying altitude and tree age conditions.

The results will help improve our understanding of the growth adaptation mechanisms of coniferous species to temperature and moisture gradients, systematically reveal the dynamic characteristics and regulatory pathways of xylem formation in Korean pine of different ages along an altitudinal gradient, and provide a solid theoretical basis and data support for predicting the dynamics of broadleaved Korean pine forests under climate change.

## 2 Materials and methods

### 2.1 Study area

This study was conducted in the Changbai Mountains region in northeastern China, which experiences a typical temperate continental monsoon climate. The baseline zone of the Changbai Mountains has a mean annual temperature of 3.6 °C, with the lowest average temperature of -14.15 °C in January and the highest average temperature of 20.82 °C in July. The mean annual precipitation ranges from 600 to 800 mm, with 80% occurring between May and September. The maximum precipitation belt is found between 1100 and 1800 m above sea level (a.s.l.).

The northern slope of the Changbai Mountains features a complete vertical vegetation spectrum. From low to high elevation, the zones are distributed as follows: Korean Pine Mixed Broadleaf-Conifer Forest Belt (500–1100 m a.s.l.): Sampling was conducted at three elevations within this belt: 750 m (42°24.2824’N, 128°06.1662’E), 950 m (42°16.1432’N, 128°09.2483’E), and 1150 m (42°11.4691’N, 128°11.8926’E). This belt is dominated by *Pinus koraiensis*, associated with species such as *Fraxinus mandshurica*, *Phellodendron amurense*, *Juglans mandshurica*, *Tilia amurensis*, and *Quercus mongolica*. Spruce-Fir Dark Coniferous Forest Belt (1100–1700 m a.s.l.): This belt is primarily composed of Picea jezoensis and Abies nephrolepis. *Betula ermanii* Dwarf Forest Belt (1700–2000 m a.s.l.): This belt is mainly dominated by Betula ermanii, Larix spp., and other species.

Korean pine, the target species of this study, is primarily distributed between 500 and 1300 m a.s.l. in the Changbai Mountains and is a crucial climax community constructor in this region. Its growth is sensitive to climate change, and its xylem exhibits distinct annual rings with clear boundaries between earlywood and latewood, making it an ideal material for studying tree-growth-environment relationships (Qian et al., 2023).

### 2.2 Sample collection and processing

From March to October 2024, sample collection was carried out at three different elevations (750 m, 950 m, and 1150 m) on the northern slope of the Changbai Mountains. Within a 30 m × 30 m quadrat at each elevation, the diameter at breast height (DBH) of trees was measured using a diameter tape to conduct a size-class survey. Trees were categorized into two size-class groups: large-diameter Korean pines (DBH: 55–65 cm) and small-diameter Korean pines (DBH: 30–35 cm).

In each plot, approximately 10 trees per diameter class were cored (two cores per tree) to determine tree age, in order to avoid large age differences among trees with similar DBH. Sampled trees were healthy, with no substantial stem or crown damage or other abnormalities. Across elevations, the ages of large-diameter pines were concentrated at 140–150 years, and those of small-diameter pines at 100–110 years (**Table 1**). Four healthy individuals with similar ages and consistent DBH were selected from each diameter class at each elevation for subsequent analysis, ensuring uniform growth conditions as much as possible. According to the Chinese National Forestry Industry Standard “Age class and Age group Classification for Major Tree Species” (LY/T 2908-2017), for natural Korean pine forests, each age class spans 20 years; trees aged 101–120 years are classified as near-mature forest, while trees aged 121–160 years are classified as over-mature forest. Therefore, according to this standard, the 100–110 year old trees in our study belong to the near-mature forest category, whereas the 140–150 year old trees belong to the over-mature forest category. Hereafter, the terms ‘large-age’ Korean pine and ‘small-age’ Korean pine refer to over-mature and near-mature Korean pine, respectively.

**Table 1:** Sampling site information of *Pinus koraiensis* in different elevations and age class in Changbai Mountains of China (Mean temperature and total precipitation in the table are growing-season data from March to October 2024.).

Sites	Age class	Elevation (m)	Mean temperature (°C)	Total precipitation (mm)	Age (year)	DBH (cm)
High elevation	Large	1150	10.28	828.98	147 ± 7	52.1 ± 2.0
	Small				107 ± 3	34.3 ± 1.4
Middle elevation	Large	950	11.82	778.73	143 ± 11	50.8 ± 5.2
	Small				103 ± 5	34.7 ± 0.9
Low elevation	Large	750	13.07	739.20	146 ± 7	53.7 ± 1.6
	Small				101 ± 4	32.5 ± 2.1

From March to October 2024, a long-term monitoring program was conducted with the following sampling frequency: 1–2 times per week during the early growing season (late March to late April), once every 15 days from May to September, and once per week during the late growing season (late September to late October).Using an increment borer, 3–4 short cores (3–5 cm in length) were extracted from each sample tree at breast height. The cores were immediately placed in FAA fixative solution (70% Ethanol: Acetic Acid: Formaldehyde = 18: 1: 1). Cores from the same tree were taken 2–3 cm apart, using a borer with a diameter of 5.15 mm. The collected cores contained tissues including the phellem, phloem, vascular cambium, and secondary xylem from the current growth period. To minimize the impact of previous sampling on subsequent collections, a staggered principle was followed: the next row of sampling points was positioned approximately 3 cm below the midpoint between every two previous sampling holes in the row above, while maintaining the same direction.

After collection, samples were processed in the laboratory. High-quality permanent sections were prepared using a sliding microtome. The sections underwent a series of steps including cleaning, staining, dehydration, rehydration, mounting, and pressing to obtain high-resolution and clear sections. Microscopic observation and photography of the anatomical structures were then performed.

**Figure 1:**
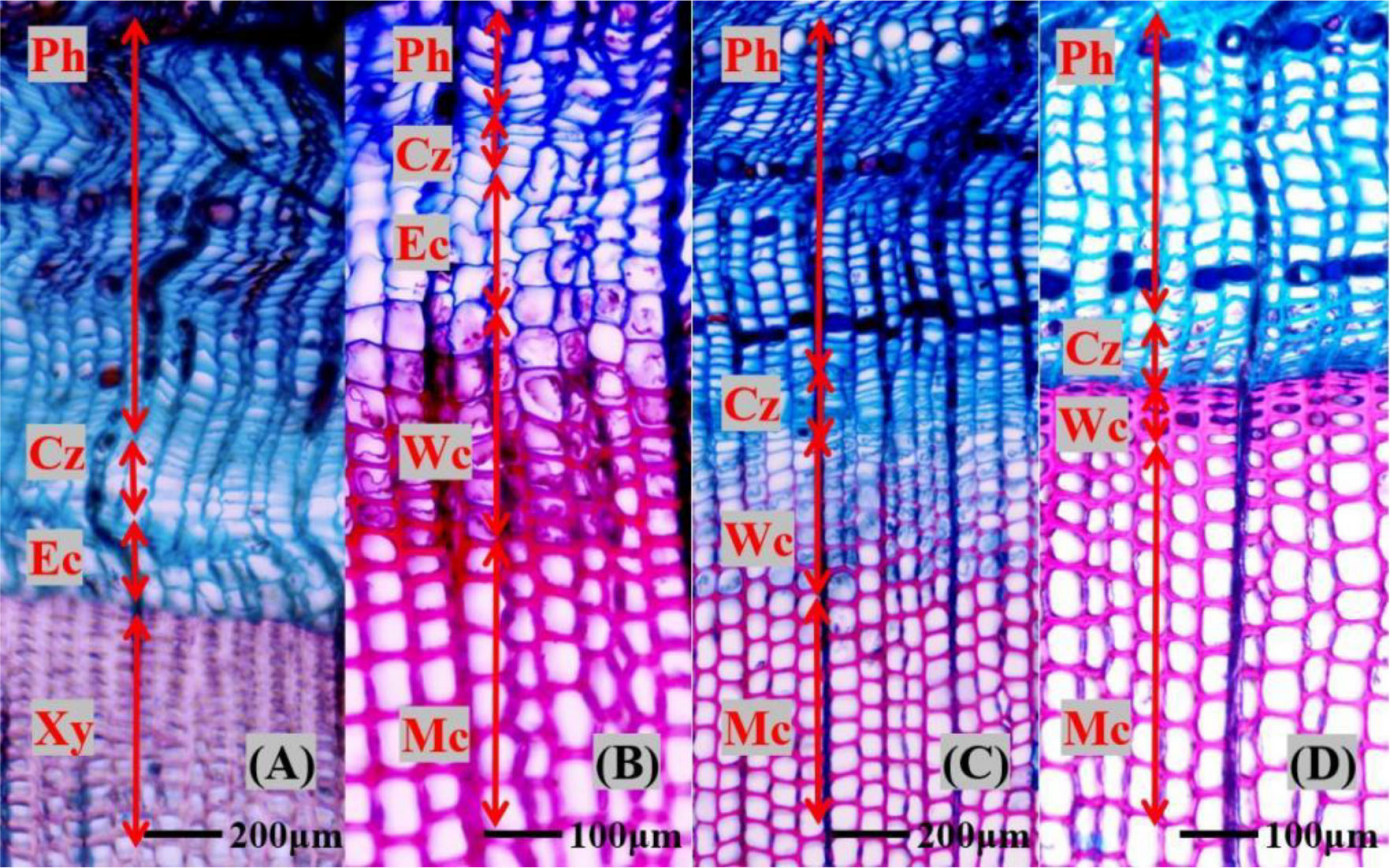
Xylem anatomical photographs of *Pinus koraiensis* in different months. Ph, phloem; Cz, Cambial zone; Xy, xylem cells; Ec, enlarging cells; Wc, wall thickening cells; Mc, mature cells. **(A)** April 14th, **(B)** June 22th, **(C)** August 13th, **(D)** September 22th.

Cambial cells (10–15 μm) were tightly arranged in radial rows and stained bluish-green. Enlarging cells (20–30 μm) retained this stain. Secondary wall thickening cells had thicker walls; still bluish-green due to incomplete lignification, but thickening walls appeared bright under polarized light (**Figure 1**).

Fully mature xylem cells, due to lignin deposition, displayed a typical purplish-red staining characteristic (Qian et al., 2023), as shown in **Figure 1**. All microscopic images were digitally captured and analyzed using Image-Pro Plus 6.0 software.

### 2.3 Data processing

#### 2.3.1 Spatial interpolation of meteorological data (950 m a.s.l.)

Daily meteorological data (temperature and precipitation) at 750 m and 1150 m a.s.l. were recorded directly by small automatic weather stations installed at these elevations (**Table 1** for the values), with a logging interval of 30 minutes. In this study, measured sites were only established at 750 m and 1150 m. Given the limited number of elevation samples, we interpolated meteorological data at 950 m using a linear regression method based on the environmental lapse rate of temperature and a simplified co-kriging method, respectively. The selection of these methods is supported by the literature (Zhu et al., 2020; He et al., 2019; Zhang et al., 2021b).

Air temperature was estimated using a linear regression model based on the temperature lapse rate, consistent with the PRISM/MRM framework recommended for complex terrain and small sample sizes (He et al., 2019; Zhu et al., 2020). The formulas are as follows:

(1) $$\text{Elevation difference: }\Delta H=H_{1150}-H_{750}$$

In Equation 1:△*H* is the elevation difference (unit: m); *H*_1150_ is the elevation value at 1150 m; *H*_750_ is the elevation value at 750 m. Substituting the numerical values yields a fixed elevation difference.

(2) $$\text{Temperature lapse rate: }r=\frac{T_{750}-T_{1150}}{\Delta H/100}$$

In Equation 2: *r* is the temperature lapse rate per 100 m increase in elevation (°C/100m); *T*_750_ is the measured temperature at 750 m elevation (°C); *T*_1150_ is the measured temperature at 1150 m elevation (°C); Δ*H* is the elevation difference.

Construct a linear regression model between temperature and elevation (Equation 3):

(3) T=a+b×H

In Equation 4: *T* is the calculated air temperature (°C); *H* is the elevation that the site will predict temperature (m); The parameter *a* is the regression intercept; *b* is the regression coefficient.

(4) $$a=\;\bar{T}-b\times \bar{H}$$

In Equation 4: $\bar{T}$ is the mean of measured temperatures; $\bar{H}$ is the mean of measured elevations.

(5) $$b=-\frac{r}{100}$$

In Equation 5: The b is the decreased temperature per meter increase in elevation (°C/m).

Precipitation was interpolated using a simplified co-kriging method with elevation as a covariate, which performs best for precipitation estimation in limited-sample situations (Zhu et al., 2020; Zhang et al., 2021b).

The distance from 950 m to both 750 m and 1150 m is equal(200 m), yielding equal weights (Equation 6):

(6) $$W_{750}=W_{1150}=0.5$$

Estimated precipitation at 950 m (Equation 7):

(7) $$P_{950}=W_{750}P_{750}+W_{1150}P_{1150}$$

Using the cross-validation method, the temperature and precipitation values at one of the elevations were back-calculated fromthe measured data at 750 m and 1150 m, and the errors were calculated using the following formula to verify the reliability of the method (Equations 8–11):

(8) $$AE=T_{\text{measured}}-T_{\text{backcalculated}}$$

(9) $$RE=\frac{T_{\text{measured}}\;-T_{\text{backcalculated}}}{T_{\text{measured}}}$$

(10) $$AE=P_{\text{measured}}\;-P_{\text{backcalculated}}$$

(11) $$RE=\frac{P_{\text{measured}}\;-P_{\text{backcalculated}}}{P_{\text{measured}}}$$

Acceptance criteria: According to the literature (He et al., 2019; Zhu et al., 2020), for temperature interpolation, the absolute error (*AE*) should be ≤ 0.5 °C and the *RE* ≤ 3%; for precipitation interpolation, the *RE* should be ≤ 15%. The results of this study met these criteria.

#### 2.3.2 Tree age determination and phenology analysis

The collected long tree-core samples from different diameter classes were transported to the laboratory and preprocessed following conventional dendrochronological procedures, including mounting, air-drying, and polishing of Korean pine tree-ring cores. Polishing was carried out sequentially using sandpaper with increasing grit sizes until tracheids and tree-ring boundaries were clearly distinguishable under a stereomicroscope. Visual cross-dating of each sample was performed under the microscope using the skeleton-plot method. Tree age groups were then assigned for subsequent analyses (Stokes and Smiley, 1996).

Based on the section images, key dates of xylem formation (expressed as Day of Year, DOY) were recorded. A digital image analysis system was used for the quantitative analysis of microscopic sections to determine key phenological time points in the xylem formation process. The specific parameters measured included: the onset of cambial activity, the onset of cell enlargement, the onset of cell wall thickening, the onset of cell maturation, the end of cell enlargement, the end of cell wall thickening, and the end of maturation (all in DOY). The criteria for identifying each developmental stage were as follows: the start of a stage was defined as the first date when at least one cell was observed entering that stage, and the end of a stage was defined as the date when cells in that stage were no longer observed in consecutive observations.

The duration of cell enlargement was the period from the start to the end of enlarging cell production. The duration of cell wall thickening was the period from the start to the completion of cell wall thickening. The total duration of xylogenesis (i.e., the xylem growth season) was defined as the period from the start of enlarging cell production to the end of cell wall thickening (Rathgeber et al, 2011).

Independent samples t-tests were used to analyze differences in xylem formation phenology of Korean pine across different age classes and elevations, while two-way ANOVA was simultaneously applied to evaluate the main effects of elevation, age class, and their interactive effects on the variables concerned.

#### 2.3.3 Xylem anatomical trait measurement

The software ROXAS (von Arx and Carrer, 2014) was used to automatically or semi-automatically read and measure xylem anatomical characteristics. This software, based on Image-Pro Plus, automatically identifies tree rings and cell boundaries, efficiently calculating bulk data parameters including tree-ring width (MRW), tracheid number (TN), tracheid diameter (Dt), and cell wall thickness (CWT), as shown in **Table 2**. Mature tracheids were classified into three different wood types: earlywood, transition wood, and latewood. According to Mork’s criterion, the classification was calculated as four times the tangential cell wall thickness divided by the radial lumen diameter. Tracheids were classified as follows: MC ≤ 0.5 for earlywood; 0.5< MC< 1 for transition wood; and MC ≥ 1 for latewood (Cuny et al., 2019).

**Table 2:** Comparison of main anatomical characteristics between large-age and small-age Korean pines at different elevations.

Location	Elevation	Tracheid diameter/ μm (Mean ± SD)		Tracheid wall thickness/ μm (Mean ± SD)	
		Large	Small	Large	Small
Earlywood	750m	31 ± 4	28 ± 3	3.5 ± 0.9	3.0 ± 0.3
	950m	30 ± 2	28 ± 2	3.8 ± 0.5	3.1 ± 0.5
	1150m	27 ± 3	23 ± 4	3.9 ± 0.7	3.4 ± 0.2
Transition area	750m	29 ± 5	27 ± 2	4.1 ± 0.4	3.8 ± 0.3
	950m	28 ± 2	26 ± 1	4.2 ± 0.5	3.9 ± 0.4
	1150m	26 ± 4	24 ± 1	4.1 ± 0.4	3.9 ± 0.3
Latewood	750m	18 ± 1	18 ± 1	4.2 ± 0.2	4.2 ± 0.1
	950m	17 ± 2	17 ± 1	4.8 ± 0.3	4.7 ± 0.7
	1150m	17 ± 1	17 ± 1	5.2 ± 0.6	5.1 ± 0.2

Based on the determined numbers of earlywood, transition, and latewood cell layers for each tree, we classified the cell layers during the developmental stages of cell enlargement, cell wall thickening, and maturation, and further examined the dynamics of earlywood, transition, and latewood tracheids across these stages.

#### 2.3.4 Radial growth quantification and standardization

The software ImageJ was used to measure the incremental growth (ring width) in the regions corresponding to cambial cells, enlarging cells, wall-thickening cells, and mature cells. For each section, three radial cell files were selected to measure the number of cell layers at each developmental stage and tree-ring width. The ring width of the newly formed xylem cells for the current year was standardized based on the previous year’s ring width using the (Equation 12):

(12) $$W_{\text{sd}}=W_{\text{i}}\times W_{\text{Pall}}/W_{\text{pi}}$$

Where: *W*_sd_ is the standardized ring width for a specific sample from a specific tree. *W_i_* is the actual measured ring width of that sample. *W_Pall_* is the average ring width of the previous year from all samples of that tree. *W_pi_* is the previous year’s ring width of the current sample.

After standardization, the sum of the growth increments in the enlarging cell, wall-thickening cell, and mature cell regions was calculated as the cumulative radial xylem growth for each sample tree. The average of all sample trees was taken to represent the xylem growth situation for the species.

#### 2.3.5 Growth curve fitting and rate calculation

The Gompertz model can generally describe the relationship between growth and time well and has been widely used in studies of xylem formation dynamics (Rossi et al., 2003). In this study, the Gompertz model was used to fit the cumulative xylem growth of individual trees and the mean cumulative xylem growth of Korean pine. Based on the fitted parameters, the date of maximum growth rate (*t*_maxr_), and the maximum daily growth rate (*r*_max_). Independent samples t-tests were used to analyze differences in the fitted parameters of Korean pine. The Equations 13–15 are as follows:

(13) $$W=Ae^{-e^{\beta -kt}}$$

(14) $$t_{maxr}=\beta /k$$

(15) $$r_{max}=kA/e$$

Where in the Gompertz model context, *W* represents total growth width; *t* is time (DOY); *A* represents the maximum growth asymptote (total growth); and *β* and *k* are the intercept parameter on the x-axis and the rate parameter, respectively.

To investigate the response of radial growth rate to the elevation gradient, the daily xylem growth rate for each sample tree of different age classes was calculated based on measured data using the (Equation 16):

(16) $$r_{i}=\left(w_{i}–\;w_{i–1}\right)/\left(t_{i}–t_{i–1}\right)$$

Where: *r_i_*is the xylem growth rate on the *i*^th^ sampling day. *w_i_* and *w_i–1_* are the cumulative xylem widths on the *i*^th^ and (*i*-1)^th^ sampling days, respectively. *t_i_* and *t*_*i*–1_ are the ith and (*i*-1)^th^ sampling dates (DOY), respectively.

#### 2.3.6 Tracheid development rate and duration proportion analysis

To explore the influence of cell change rate and duration proportion during the cell enlargement and cell wall thickening phases on tracheids, the tracheid enlargement rate, wall thickening rate, and duration proportion for earlywood, transition, and latewood were calculated for Korean pines of different age classes at each elevation, based on measured data. The Equations 17–22 were as follows:

(17) $$\begin{array}{c} \text{Earlywood Tracheid Growth Rate}\\ =(\text{Difference in width during enlargement}/\\ \text{wall thickening stage between sampling days})/\\ \left(\text{Number of days between two sampling dates}\right) \end{array}$$

(18) $$\begin{array}{c} \text{Earlywood Tracheid Duration Proportion}=\\ \left(\text{Earlywood end date}–\text{Earlywood start date}\right)\\ /\left(\text{Total phase duration in days}\right) \end{array}$$

(19) $$\begin{array}{c} \text{Transition Wood Tracheid Growth Rate}\\ =(\text{Difference in width during enlargement}/\\ \text{wall thickening stage between sampling days})/\\ \left(\text{Number of days between two sampling dates}\right) \end{array}$$

(20) $$\begin{array}{c} \text{Transition Wood Tracheid Duration Proportion}\\ =(\text{Transition wood end date}–\text{Transition wood start date})/\\ \left(\text{Total phase duration in days}\right) \end{array}$$

(21) $$\begin{array}{c} \text{Latewood Tracheid Growth Rate}\\ =(\text{Difference in width during enlargement}/\\ \text{wall thickening stage between sampling days})/\\ \left(\text{Number of days between two sampling dates}\right) \end{array}$$

(22) $$\begin{array}{c} \text{Latewood Tracheid Duration Proportion}\\ =\left(\text{Latewood end date}–\text{Latewood start date}\right)/\\ \left(\text{Total phase duration in days}\right) \end{array}$$

In Equations 17, 19, 21, the Earlywood, Transition Wood, and Latewood Tracheid Rates refer to the enlargement or wall thickening rate of the respective tracheid type on the *n*^th^ sampling day. The “Difference in width during enlargement/wall thickening stage between sampling days” refers to the change in width of the earlywood, transition wood, or latewood zone during the enlargement or wall thickening phase between two consecutive sampling dates.

In Equations 18, 20, 22, the Earlywood, Transition Wood, and Latewood Tracheid Duration Proportion refers to the proportion of the duration of the enlargement or wall thickening phase occupied by the respective tracheid type within the total duration of that specific phase (enlargement or wall thickening). “Earlywood end date – start date” refers to the duration from the earliest start to the latest end of the enlargement/wall thickening for earlywood tracheids collectively (as a group, not individual cells). The same logic applies to transition and latewood tracheids.

A sample size of four trees per age class was used in this study, which conforms to the general standards in studies of tree xylem formation and cambial phenology. In studies involving high-frequency continuous sampling and high-resolution anatomical observations, 3–5 replicates per group have been proven to reliably reflect the typical physiological rhythms of the species (e.g., Li S. et al., 2024). The calculated means and standard errors (presented as error bars) clearly characterize the variability of the data. Data analysis was performed using SPSS and R 4.1.3 software. Graphs were created using Origin.

## 3 Results

### 3.1 Phenological differences in xylem formation of Korean pine across altitudes and age classes

**Figure 2:**
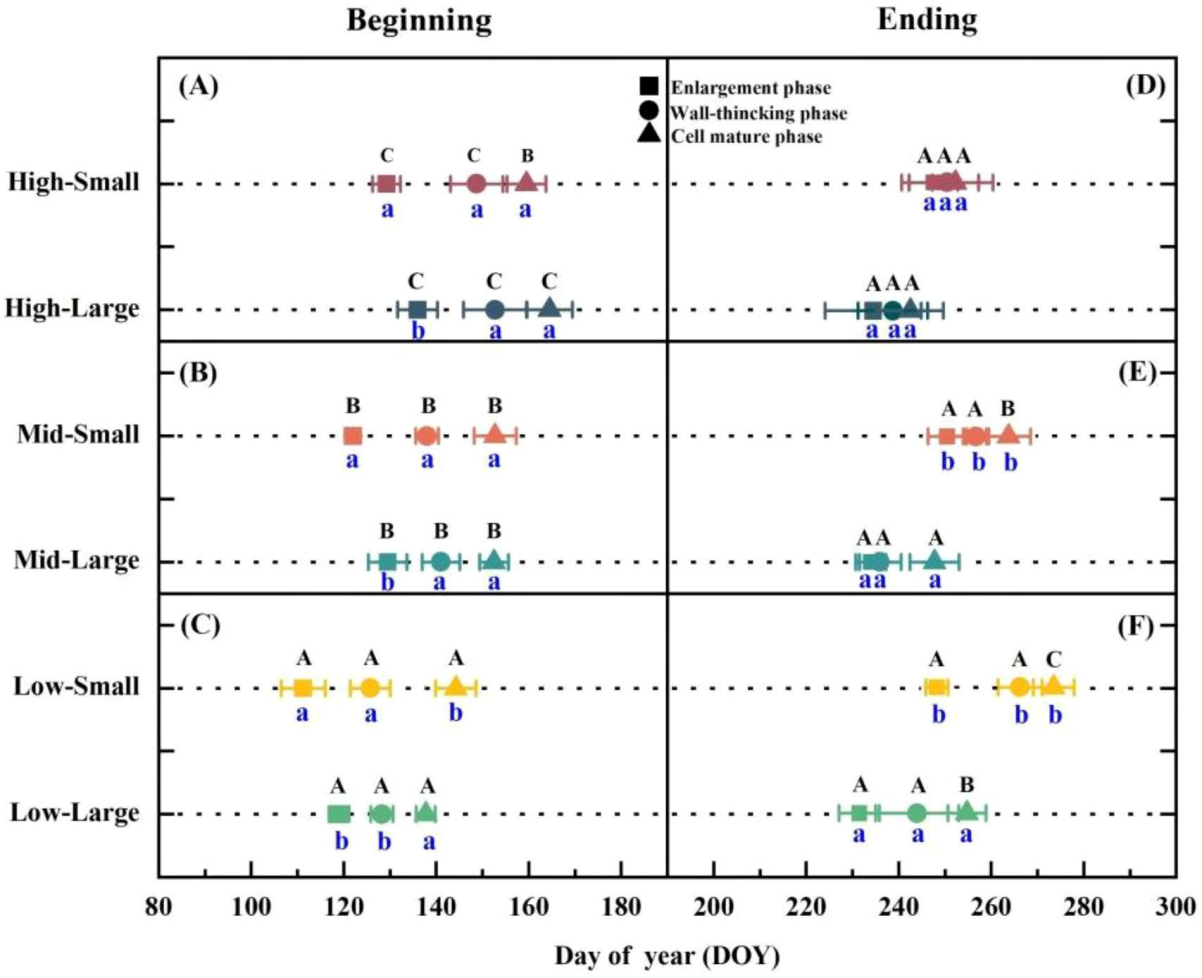
Comparison of the beginning **(A–C)** and ending **(D–F)** dates of xylem growth in *Pinus koraiensis* of different altitudes and age grades. In this study, beginning denotes the first observed occurrence of cambial cell enlargement (solid square), secondary cell wall thickening (solid circle), and cell maturation (solid triangle) accompanied by lignification, while Ending denotes the last observed occurrence of these respective processes. Uppercase letters indicate significant differences among elevations, and lowercase letters indicate significant differences among age classes. Significant differences are indicated by uppercase and lowercase letters to denote statistical comparisons among different factors. Uppercase letters (A, B, C) represent significant differences among the three elevational sites (High, Mid, Low) within the same age class. Lowercase letters (a, b) represent significant differences between the two age classes (Small, Large) within the same elevational site. Different letters indicate significant differences at the *p* < 0.05 level.

Pronounced differences in xylem formation phenology were observed between altitude levels and tree age classes (**Figure 2**). At 750 m altitude, small-age trees initiated cell enlargement earlier (April 20^th^, DOY 111 ± 5) than large-age trees (April 28^th^, DOY 119 ± 3), resulting in an 8-day earlier start and a total enlargement duration approximately 17 days longer (ending September 4^th^ vs. August 18^th^) (**Figures 2C, F**). For cell wall thickening, the onset time did not differ significantly between tree age classes, but small-diameter trees completed it later (September 22^nd^ vs. August 31^st^). The maturation phase began about 6 days later in small-age trees (May 23^rd^ vs. May 17^th^) but ended significantly later (September 29^th^ vs. September 10^th^). Overall, large-age trees completed seasonal growth about 19 days earlier, resulting in an average growth period roughly 27 days shorter (**Figures 2C, F**).

Across all altitudes, small-age trees consistently initiated developmental phases earlier, with statistically significant differences in cell enlargement onset (**Figures 2A–C**). Phase completion was also generally later for small-age trees, with significant differences at most altitudes (**Figures 2D–F**).

Across all age classes, Korean pine was significantly affected by elevation. Specifically, for every 200 m increase in elevation, the onset of the cell enlargement, wall thickening, and cell maturation phases was delayed by an average of 10–12 days, whereas the ending of each phase advanced by an average of 8–12 days. Consequently, the duration of the entire developmental process shortened by 12–19 days with increasing elevation.

### 3.2 Seasonal dynamics of xylem formation

**Figure 3:**
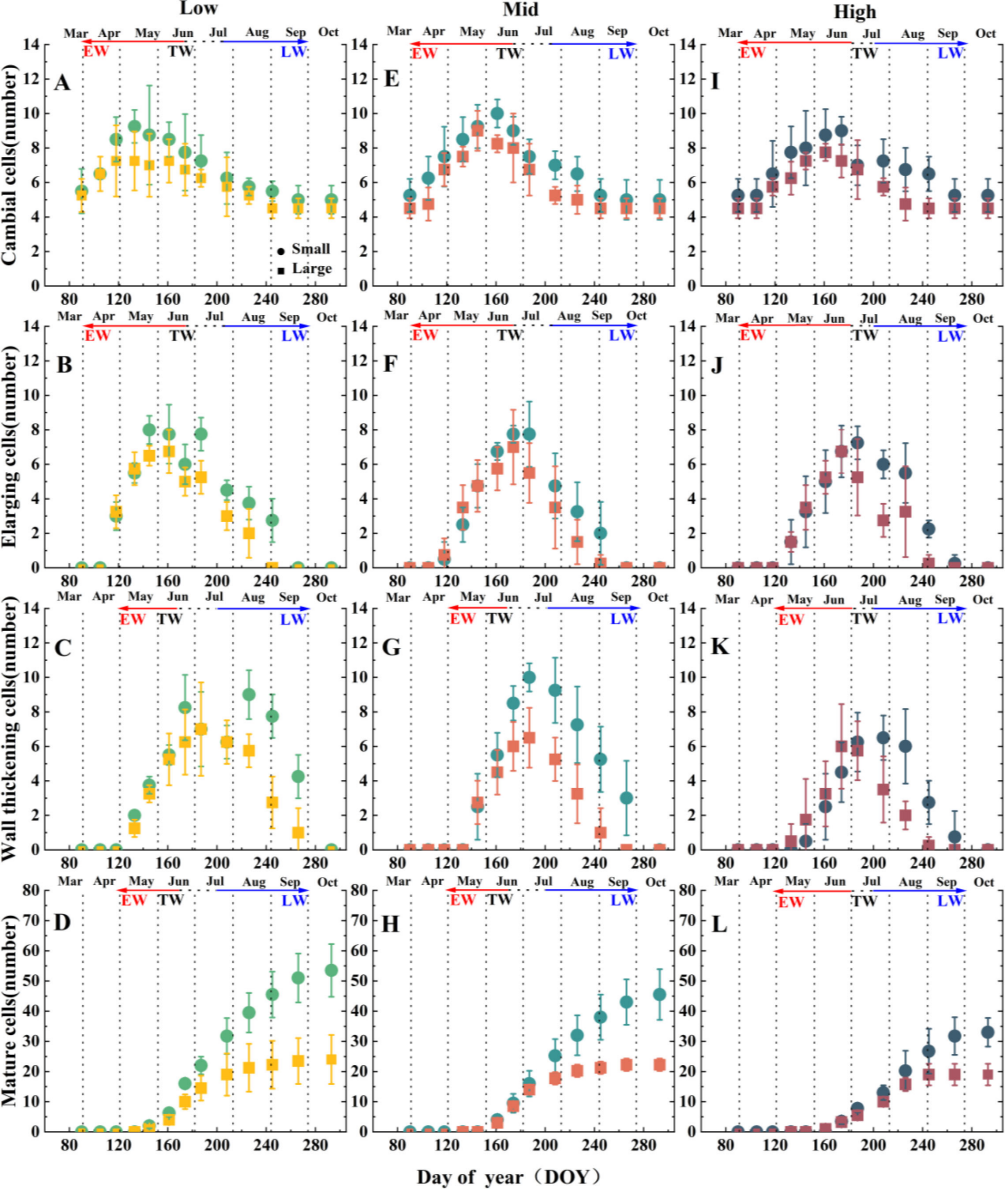
Comparison of the number of cell layers in the cambium **(A, E, I)**, xylem cell enlargement zone **(B, F, J)**, cell wall thickening zone **(C, G, K)**, and mature cell zone **(D, H, L)** of *Pinus koraiensis* at different altitudes and age grades. The solid circle denotes the small-age class of *Pinus koraiensis*, and the solid square denotes the large-age class. EW, Earlywood; TW, Transition area; LW, Latewood. Low, low elevation; Mid, middle elevation; High, high elevation.

In this study, during the early growing season, the cambium of Korean pine (*Pinus koraiensis*) consisted of only 4 to 6 cell layers, with no significant altitudinal variation, although small-age trees had 1–2 fewer layers than large trees. Cambial division was active from April to June (**Figure 3A, E, I**). At 750 m altitude, division began on April 2^nd^ (DOY 93 ± 11), with xylem formation starting 18 days later on April 20^th^ (DOY 111 ± 5). Each 200 m altitude increase delayed initiation by about 13 days (**Figures 3A, E, I**).

Cambial activity ceased before August 20th (DOY 232 ± 7) at 750 m. While the phenology of xylem differentiation stages (enlargement, wall thickening, maturation) exhibited significant altitudinal variation, the cessation timing of cambial cell division showed no significant differences across elevations. However, small-age trees started activity 5–7 days earlier and ended 8–16 days later than large trees, extending their total growth duration by 16–27 days.

At 750 m and 950 m, earlywood, transition wood, and latewood formation followed similar timelines: earlywood from April to June, transition from June to early July, and latewood from July to September. At 1150 m, the earlywood-to-latewood transition period was significantly shorter (*P* < 0.05), lasting only 21 ± 5 days—14 and 13 days shorter than at 750 m (35 ± 4 days) and 950 m (34 ± 3 days), respectively (**Figures 3A–L**).

The active periods for changes in xylem cell layer numbers were consistent across altitudes and age: enlarging cells peaked from May to July (**Figures 3B, F, J**), wall-thickening cells from June to August (**Figures 3C, G, K**), and mature cells from July to September (**Figures 3D, H, L**). Small-age trees showed a declining trend in mature cell numbers with altitude, while large trees exhibited developmental delays (**Figures 3D, H, L**). Large trees generally had fewer cell layers across developmental stages.

In summary, Korean pine xylem development is jointly regulated by altitude and tree age, with May–September as the key developmental window, and small trees at high altitudes showing reduced mature cell production.

### 3.3 Fitting of intra-annual xylem growth dynamics

**Figure 4:**
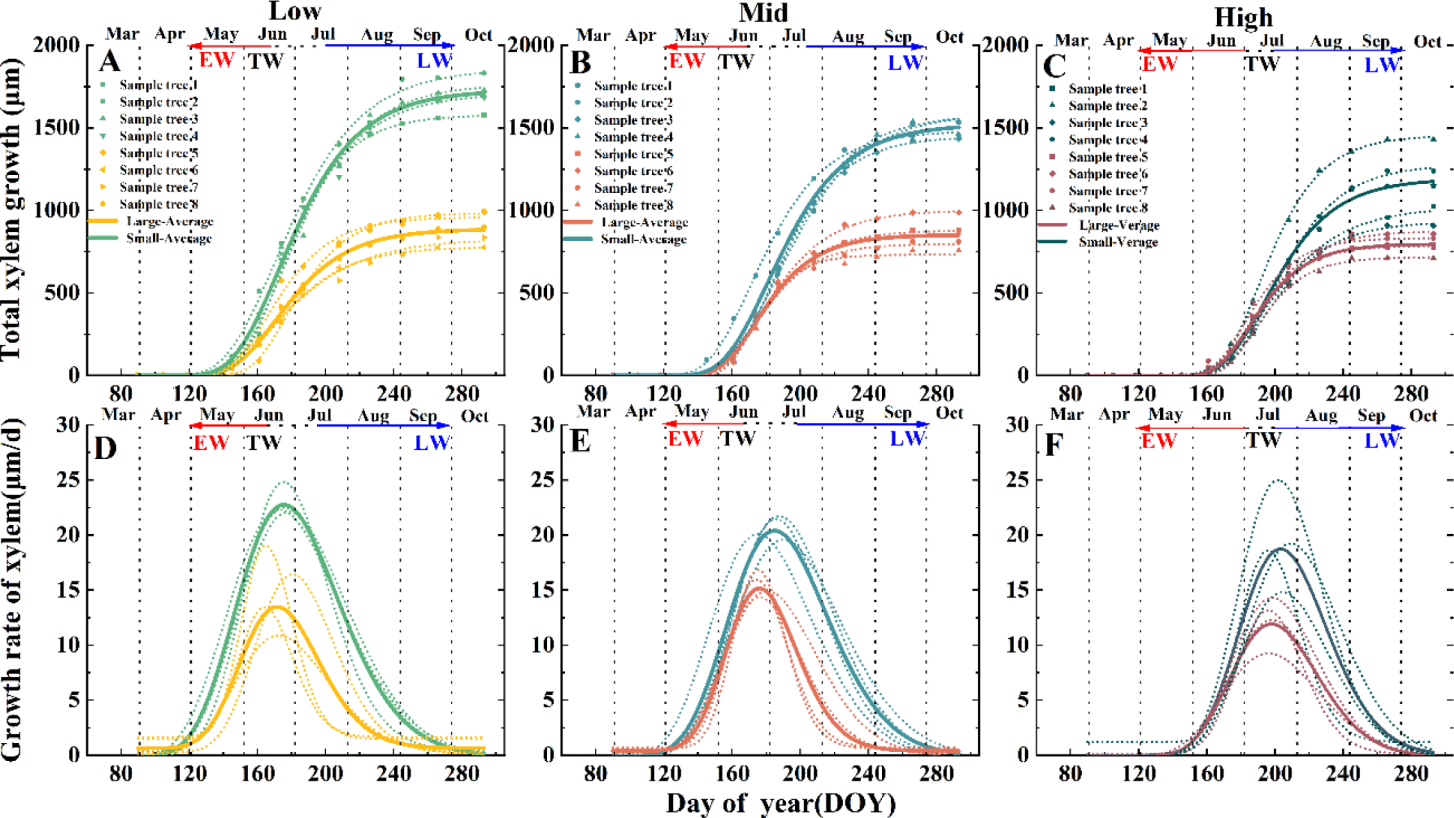
Gompertz model fitted for accumulated xylem growth **(A–C)** and growth rates **(D–F)** of *Pinus koraiensis* at different elevations. Low elevation: **(A, D)**; Middle elevation: **(B, E)** High elevation: **(C, F)**. EW- earlywood, TW, transition area, LW, latewood.

This study applied the Gompertz model to analyze the xylem growth dynamics of *Pinus koraiensis* across different elevations (750 m, 950 m, and 1150 m) (**Figure 4**). By fitting the cumulative xylem growth increment of individual trees and their means, the model demonstrated strong explanatory power, with adjusted R² values mostly exceeding 0.85 for most trees and reaching above 0.95 and 0.98 for mean growth increments, effectively capturing the species’ xylem growth dynamics.

In this study, *t*_maxr_ and *r*_max_ were obtained by fitting the Gompertz model to observed data, in which xylem growth increment was the observed data. Distinct differences in growth parameters were observed along the elevation gradient. At 750 m, small-age trees showed the greatest growth potential, with the maximum growth rate occurring on day 172, while at 1150 m, their growth potential decreased significantly. Overall, each 200 m elevation increase reduced total xylem growth by 278 μm in large-age trees and 54 μm in small-age trees.

With increasing elevation, the timing of the maximum growth rate gradually delayed. The inflection point for small-age trees at 1150 m was delayed by about 20 days compared to 750 m, and by 14 days for large-age trees. The maximum growth rate of small-age trees decreased with elevation, whereas no significant difference was observed in large-age trees. Additionally, the reduction in total xylem growth was more pronounced in small-age trees (**Figures 4A–F**).

### 3.4 Tracheid development

Altitude and tree age class significantly and independently regulated the wood formation process of *Pinus koraiensis* (all interactions *P* > 0.05, **Table 3**). During cell enlargement, altitude significantly affected earlywood tracheid diameter (*F* = 5.745, *P* = 0.012) and transition area expansion rate (*F* = 5.708, *P* = 0.018). Tree age significantly influenced the duration proportion of earlywood enlargement (*F* = 5.745, *P* = 0.028) and earlywood tracheid diameter (*F* = 6.719, *P* = 0.018). Latewood tracheid parameters showed no significant response to the altitude-size interaction (*P* > 0.05).

**Table 3:** Two factor analysis of variance on the effects of elevation and age on the growth rate, duration, and tracheid diameter of early and late wood and their transition area of *Pinus koraiensis* xylem during cell enlargement period.

Location	Parmeter of cell enlargement period	Elevation		Age class		Elevation × Age class	
		*F*	*P*	*F*	*P*	*F*	*P*
Earlywood	Growth rate	**3.20**	**0.05**	3.51	0.07	0.24	0.79
	Duration time	2.00	0.16	**5.75**	**0.03**	0.14	0.87
	Tracheid diameter	**5.75**	**0.01**	**6.72**	**0.02**	0.38	0.69
Transition area	Growth rate	**5.71**	**0.02**	3.32	0.07	0.04	0.96
	Duration time	0.11	0.89	0.07	0.80	1.00	0.39
	Tracheid diameter	2.73	0.09	1.29	0.27	0.12	0.89
Latewood	Growth rate	1.34	0.30	2.22	0.16	0.08	0.94
	Duration time	1.39	0.28	0.15	0.70	0.10	0.91
	Tracheid diameter	0.64	0.54	2.30	0.15	0.14	0.87

The bold values represent statistically significant differences at P < 0.05 level.

**Figure 5:**
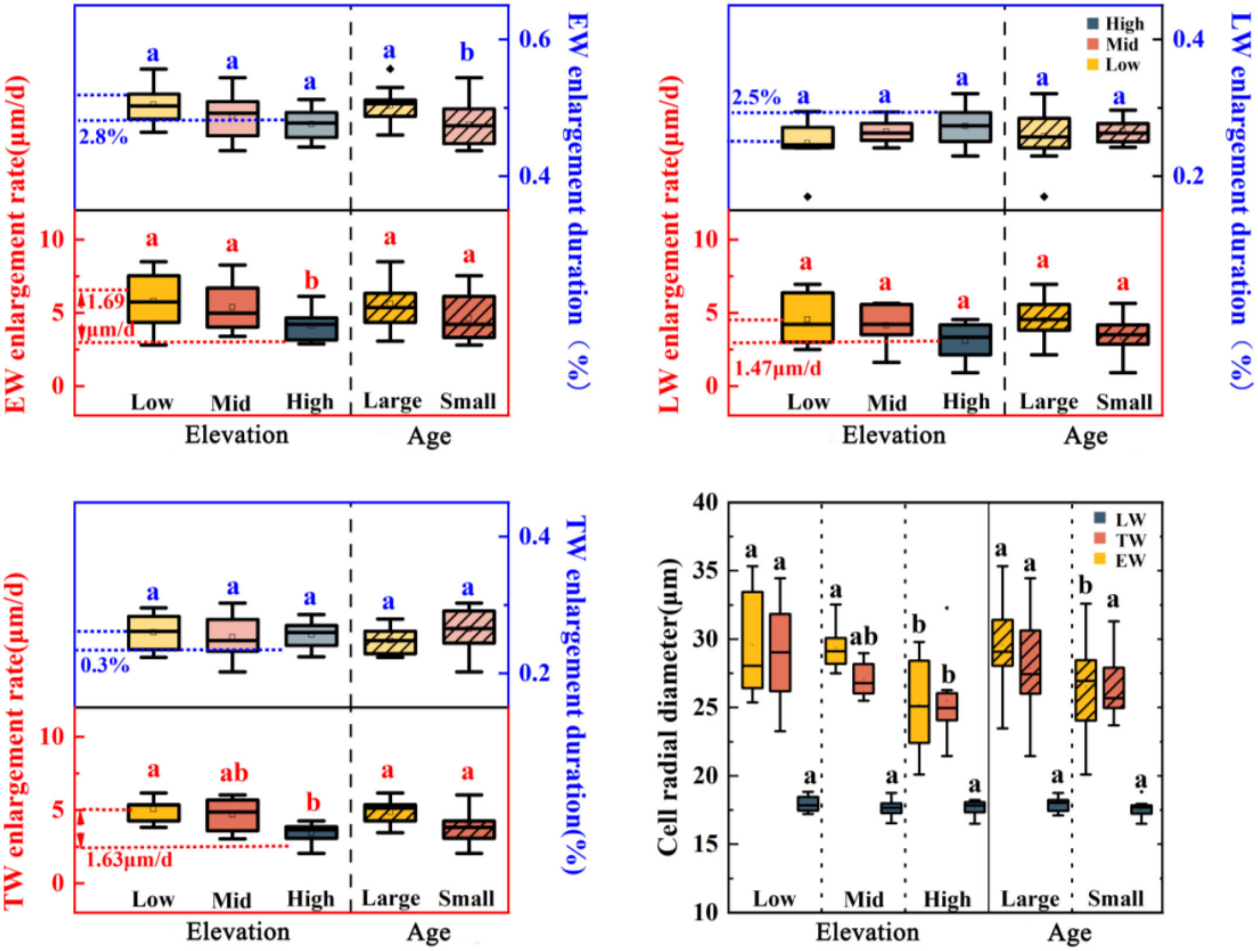
Comparison of cell growth rate, duration, and tracheid diameter among earlywood (EW), latewood (LW) and their transition (TW) of *Pinus koraiensis* during the cell enlargement stage at different altitudes and age grades. The lowercase letters indicate significant differences among age classes (large and small age) or elevations (low, middle and high elevation).

Along the altitudinal gradient, while duration allocation among wood types remained unchanged (*P* > 0.05, **Figure 5**), the average expansion rates of earlywood and transition area decreased significantly with elevation (by 1.69 μm/d and 1.63 μm/d respectively, *P* < 0.05). Consequently, earlywood and transition area tracheid diameters at 1150 m were 4.36 μm and 3.5 μm smaller than at 750 m, while latewood showed only slight, non-significant reduction.

Larger trees had a 2.7% shorter earlywood enlargement phase (P< 0.05), similar expansion rates to smaller trees (*P* > 0.05), but significantly smaller earlywood tracheid diameters (by 3.07 μm).

Altitude and tree age class significantly influenced the cell wall thickening phase of *Pinus koraiensis* (**Table 4**). The process showed significant spatiotemporal heterogeneity. Altitude had highly significant effects on the duration proportion of latewood wall thickening (*F* = 7.77, *P* = 0.004) and latewood tracheid wall thickness (*F* = 18.19, *P<* 0.001), with walls 0.91 μm thicker at 1150 m than at 750 m. Tree age significantly affected earlywood wall thickening rate (*F* = 7.39, *P* = 0.017) and earlywood tracheid wall thickness (*F* = 5.01, *P* = 0.038). All “elevation × age” interactions were non-significant (*P* > 0.05), confirming independent effects.

**Table 4:** Two factor analysis of variance on the effects of elevation and age on the growth rate, duration, and tracheid wall thickness of early and late wood and their transition period of *Pinus koraiensis xylem* during cell wall thickening period.

Location	Parmeter of cell wall thickening period	Elevation		Age class		Elevation × Age class	
		*F*	*P*	*F*	*P*	*F*	*P*
Earlywood	Growth rate	2.00	0.17	0.94	0.41	0.21	0.82
	Duration time	0.25	0.78	0.15	0.71	0.54	0.59
	Tracheid wall thickness	0.06	0.94	1.40	0.26	0.60	0.56
Transition area	Growth rate	1.77	0.22	0.01	0.94	0.15	0.86
	Duration time	0.80	0.47	0.09	0.77	0.07	0.93
	Tracheid wall thickness	0.13	0.88	3.49	0.08	0.10	0.90
Latewood	Growth rate	0.11	0.90	0.12	0.74	0.48	0.63
	Duration time	**7.77**	**<0.001**	0.64	0.44	0.16	0.85
	Tracheid wall thickness	**18.19**	**<0.001**	0.19	0.67	0.05	0.95

The bold values represent statistically significant differences at P < 0.05 level.

**Figure 6:**
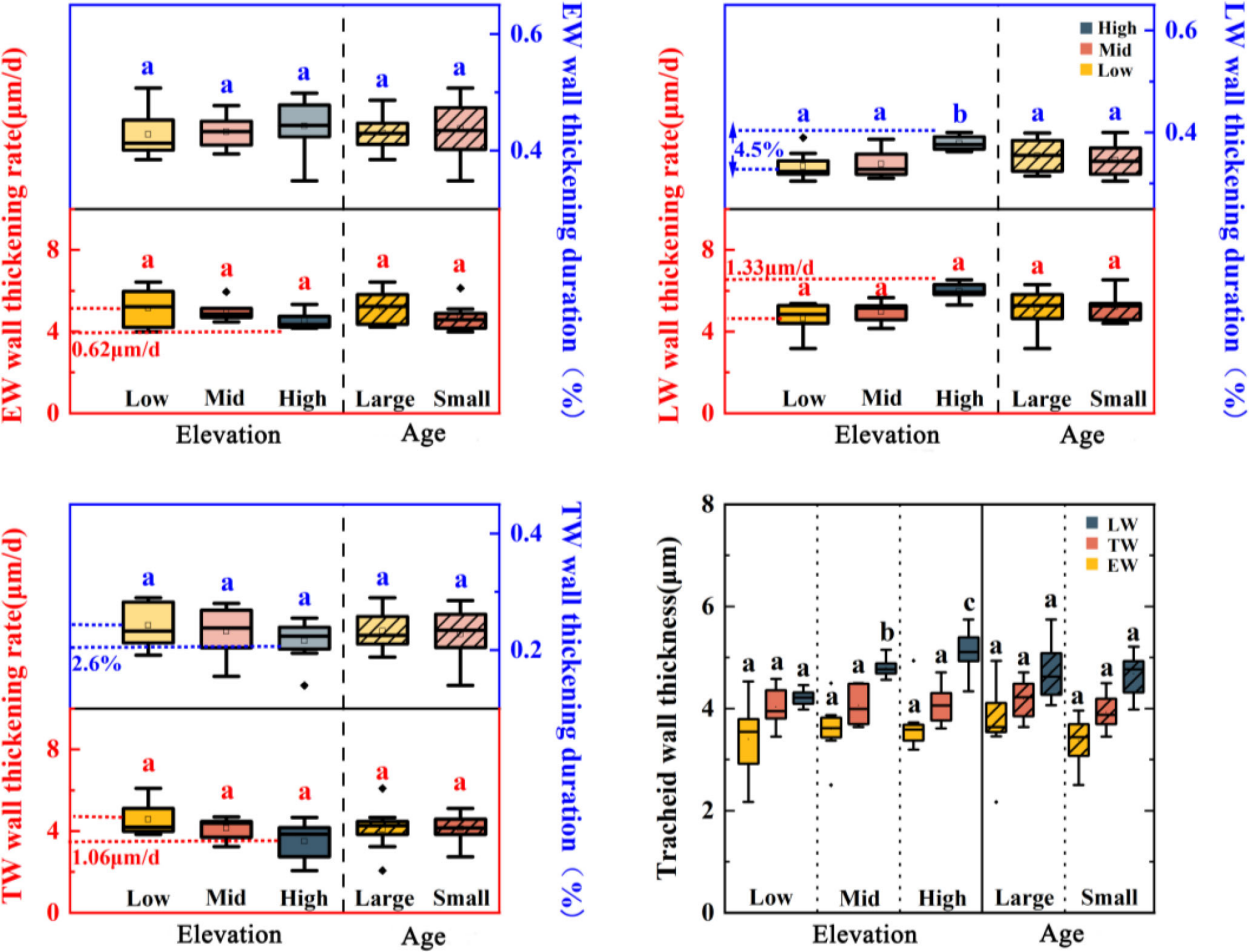
Comparison of cell growth rate, duration, and tracheid wall thickness among earlywood (EW), latewood (LW) and their transition (TW) of *Pinus koraiensis* during the cell wall thickening stage at different altitudes (low, middle and high) and age class (large and small). The lowercase letters indicate significant differences among age classes or elevations.

Along the altitudinal gradient, while earlywood and transition wood duration proportions remained stable (*P* > 0.05, **Figure 6**), the latewood phase was significantly prolonged (*P* < 0.05) with increased final wall thickness (*P* < 0.01). In contrast, age classes showed no significant differences in duration allocation or thickening rates among wood types, though larger trees had non-significantly thicker walls (*P* > 0.05).

## 4 Discussion

### 4.1 Influence of altitudinal variation on xylem formation dynamics in *Pinus koraiensis*

As elevation increases, the onset of cambial activity is delayed and the cessation of lignification occurs earlier, together shortening the duration of xylem formation in Korean pine, thereby reducing the final xylem growth increment (**Figures 2**–**4**). Although precipitation increases with elevation, the initiation of cambial cell activity is delayed across all age classes (**Figure 3**). Consistent with findings from 46 sites across the Northern Hemisphere, temperature is the key driver of the start of cambial activity in conifers (Delpierre et al., 2019); rising temperatures tend to promote earlier resumption of cambial activity and thus prolong the period of wood formation (Boulouf-Lugo et al., 2012). Climate-change-induced warming in spring can trigger an earlier reactivation of cambial activity, thereby extending the duration of wood formation (Boulouf-Lugo et al., 2012). Studies on Juniperus przewalskii in the northeastern Tibetan Plateau (Zhang et al., 2018a) and on Larix decidua in the southern French Alps (Saderi et al., 2019) have shown that the onset of cambial activity is significantly associated with temperature, occurring earlier at lower elevations and becoming progressively delayed with increasing elevation along a linear trend, which in turn postpones the onset of the successive stages of xylem formation.

For every 200 m increase in altitude, the onset of cambial activity in *Pinus koraiensis* was delayed by approximately 13 days (**Figure 3**). Lower temperatures delayed the phenological initiation of cambial reactivation at high altitudes. This trend aligns with studies on cold-temperate conifers such as European larch (*Larix decidua*) and Norway spruce (*Picea abies*) in the Alps, where increasing altitude delays the initiation of xylem formation and shortens the entire cell development cycle (Zhang et al., 2018; Fatichi et al., 2014; Miller et al., 2020; Saderi et al., 2019). The onset of cambial activity occurs progressively earlier from the top to the bottom along the altitudinal gradient (Malik et al., 2020); for instance, in *Juniperus przewalskii*, cambial activity at the highest altitude started more than one month later than at the lowest altitude (Zhang et al., 2018). Temperature influences cambial activity by regulating the supply of non-structural carbohydrates (NSC) in the phloem, which provide essential energy for cell division (Oribe et al., 2003). Temperature also modulates metabolic rates and the levels of hormones such as auxin, cytokinins, and gibberellins, thereby controlling the activity of cyclin-dependent kinases (CDKs), which are critical for the initiation of cell division (Ursache et al., 2013; Stals and Inzé, 2001).

In boreal coniferous forests, the initiation of cell enlargement is typically sensitive to temperature (**Figure 2**). For example, in Norway spruce, heating and cooling treatments can alter the rate and duration of vascular cambium cell division, thereby changing the duration of wood formation (Gricar et al., 2007). The earlier cessation of the latewood phase (secondary wall thickening and lignification) is closely related to decreased cell metabolic rates under low temperatures (Ployet et al., 2018) and impaired lignin synthesis (Antonova et al., 2014). Despite ample precipitation in the study area, lower soil temperatures at high altitudes reduce root absorption efficiency, indirectly limiting the duration of cambial activity (Qian et al., 2023; Montagnoli et al., 2019).

The transition period between earlywood and latewood in *Pinus koraiensis* shortened significantly with increasing altitude. Our data showed that the transition period length was similar at 750 m and 950 m, but was shortened by approximately 14 days at 1150 m (**Figure 3**). Similar phenomena have been reported in Norway spruce and Siberian larch (*Larix sibirica*), where low temperature at high altitudes constrains cambial phenology and alters the earlywood-to-latewood ratio (Rozenberg et al., 2020; Cuny and Rathgeber, 2016). Consistent with our observed shifts in cambial initiation and growing season duration across elevations, this pattern suggests that the delayed onset and shortened growth period at high altitudes compress the transition interval, while maintaining the essential functions of earlywood for water conduction and latewood for mechanical support. It is noteworthy that while previous studies often focused on the impact of delayed earlywood initiation on the full-season structure, this study reveals the significant role of the shortened transition period. Under climate warming scenarios, the transition period may prolong, subsequently affecting the latewood proportion.

The altitudinal gradient also influenced the kinetics of xylem cell enlargement and structural characteristics in *Pinus koraiensis*. The average expansion rate of earlywood and transition area tracheids decreased with increasing altitude, resulting in significantly smaller tracheid diameters in earlywood and transition area, while the latewood diameter only decreased slightly, albeit not significantly (**Figure 5**). Consistent with previous studies on fir in the northwestern Himalayas, the rate of cell enlargement decreased with increasing elevation (Malik et al., 2020). This is likely because low temperatures may inhibit cambial cell division, thereby reducing the rate of cell enlargement (Begum et al., 2010). The final cell structure depends on the duration and growth rate of each developmental phase (Cuny et al., 2019). In conifers, cell enlargement is the dominant phase; therefore, a shorter enlargement period and a lower enlargement rate lead to reductions in tracheid lumen area and tracheid diameter in tree rings (Buttò et al., 2019; Pittermann and Sperry, 2006; Rungwattana and Hietz, 2018).

The proportion of duration in the latewood phase and the final tracheid wall thickness in *Pinus koraiensis* were sensitive to altitude. In high-altitude regions, the duration of the latewood tracheid wall thickening phase was prolonged, the rate increased, and the wall thickness increased (**Figure 6**). This aligns with the trend observed in high-latitude or high-altitude tree species where latewood tracheids become smaller but have thicker walls (Zeng et al., 2018), suggesting that while low temperatures inhibit cell enlargement, leading to reduced diameter, the prolonged duration of wall thickening results in higher latewood density. The shortened cell enlargement period under low temperatures, coupled with a relatively extended tracheid wall thickening period, might be related to less severe water deficit stress (Pompa-García et al., 2023; Begum et al., 2010). Although the number of tracheids is limited in high-altitude *Pinus koraiensis*, structural compensation is achieved by increasing tracheid wall thickness, which helps maintain wood mechanical strength and frost resistance.

The fitting of the radial growth rate function for *Pinus koraiensis* xylem showed that growth rate decreased and xylem growth increment reduced with increasing altitude for trees of the same age class (**Figure 4**). Xylem cell growth increment is jointly influenced by growth rate and duration (Malik et al., 2020). In arid and semi-arid regions, the cell division rate affects tree adaptability, particularly when growing conditions are relatively humid, cambium cells accelerate division, and growth rate becomes the dominant factor (Rathgeber et al., 2011). These findings are consistent with our conclusion that growth rates are higher and growth increments are greater at lower altitudes (**Figure 4**).

### 4.2 Differences in xylem development of *Pinus koraiensis* across age classes

Most studies investigating the effects of altitude on wood formation have not considered the influence of tree age class on tree growth, or have only considered trees of similar age classes along the altitudinal gradient. This study found that the start dates of all developmental phases in small-age *Pinus koraiensis* were earlier than those in large-age individuals, and the end dates were generally later except at the high altitude of 1150 m, resulting in a prolonged growing season (**Figure 2**). This is consistent with heating experiments on trees of different ages, where cambial activity started earlier in 55-year-old trees compared to 80-year-old trees (Begum et al., 2010). Our study found that, under controlled external conditions such as temperature, endogenous factors (e.g., tree age) play a key role in cambial reactivation (**Figure 3**). This aligns with observations in Scots pine (*Pinus sylvestris*) and Douglas-fir (*Pseudotsuga menziesii*), where younger individuals typically exhibit longer periods of cambial activity (Li et al., 2013).

This result indicates that small-age trees have greater radial growth potential during the growing season, thereby contributing more to stand carbon sinks and wood production. Multiple studies on intra-annual radial monitoring have shown that, compared to large-age trees, younger individuals tend to initiate cambial activity earlier, have a longer growing season, and produce more xylem cells (David et al., 2024). For example, studies on *Pinus tabuliformis* in the Hasi Mountains and *Juniperus przewalskii* in the Qilian Mountains have both confirmed that younger trees exhibit earlier onset of cambial activity and faster growth rates (Zeng et al., 2018). Scholars have thoroughly explored the physiological mechanisms by which tree age regulates tree growth, and the explanation may lie in differences in resource allocation strategies: large-age trees adopt a more conservative growth strategy, allocating more resources to defense rather than growth. By delaying the onset of cambial activity, they protect meristematic tissues from spring frost damage, thereby ensuring their growth and development (Zeng et al., 2018).

The present study is primarily based on age group classification for analysis and discussion. However, tree size per se can drive the xylem formation process independently of age and is a key endogenous factor regulating cambial activity and cell differentiation. Previous studies, by rigorously distinguishing between age and size effects, have demonstrated that tree size can independently regulate xylem phenology and growth duration (Zeng et al., 2018). Even under conditions of the same tree age, significant differences exist in the onset and cessation of cambial activity and the length of the growing season among individuals of different sizes. Meanwhile, the duration of xylem cell differentiation directly determines tracheid size and wall thickness, and this differentiation duration itself exhibits significant size dependence (Buttò et al., 2019). Therefore, when selecting samples for different age groups, we strictly controlled for differences in diameter at breast height (DBH) among individuals of the same age, ensuring that the size (DBH) of individuals within the same age class remained largely consistent. This effectively eliminated the independent interference of tree size per se on the xylem formation process, ensuring that our results accurately reflect the true effect of tree age.

With increasing altitude, the initiation of all developmental phases in *Pinus koraiensis* was delayed. The end date of the maturation phase in small-age individuals advanced by 10–12 days, while in large-age individuals it advanced by only about 5 days (**Figure 2**). This differential response to altitude based on age class is consistent with studies on European larch (*Larix decidua*) in the Alps and boreal coniferous forests, where smaller individuals are more sensitive to low temperatures at high altitudes (Moser et al., 2010). The higher sensitivity of young Korean pine trees to low temperature may be attributed to differences in NSC content among trees of different ages. Hartmann et al. (2018) analyzed in detail the differences in NSC between seedlings and mature trees, noting that “The amplitude of seasonal changes in C reserves is more pronounced in tree seedlings and young saplings.” Although the young Korean pine trees in this study are not seedlings, they still differ significantly from large trees. Weber et al. (2018) also pointed out that although seedlings have very low NSC concentrations, they can survive for several days under stress conditions. However, opposite conclusions also exist: O’Brien et al. (2014) found that ten species of tropical tree seedlings had higher NSC concentrations under drought stress, thereby enhancing water potential to survive drought. This finding holds significant implications for silvicultural strategies under climate change and the regeneration of high-mountain forests, suggesting that early carbon source environment management for small-age individuals should be strengthened.

At the same altitude, small-age individuals had more xylem cell layers than large-age individuals and exhibited clear advantages across the cell enlargement, wall thickening, and maturation phases (**Figures 3**, **4**). This age-class difference aligns with the growth patterns observed in most coniferous forests across the Northern Hemisphere, where younger individuals possess higher cell production rates and denser cambial cell layers (Rathgeber et al., 2016; Cuny et al., 2015). In conifers, cell enlargement is a major phase, influencing not only ring width (Cuny and Rathgeber, 2016) but also determining the tracheid lumen area across the entire tree ring (Buttò et al., 2019). Tracheid morphology directly affects water transport capacity (Rossi et al., 2006); for instance, in *Abies fabri*, the earlywood water transport rate is about 11 times that of latewood, with over 90% of water transport originating from earlywood (Čufar et al., 2011);. In this study, large-age *Pinus koraiensis* had a shortened proportion of duration in the earlywood tracheid enlargement phase and significantly smaller earlywood tracheid diameters compared to small-age individuals, while latewood parameters were insensitive to age class (**Figures 5**, **6**). This phenomenon might be because large-age individuals tend to allocate growth resources more towards structural maintenance and resistance to external stresses rather than radial expansion (Kennedy et al., 2010; Yan et al., 2021). The insensitivity of latewood parameters in *Pinus koraiensis* to the interaction between age class and altitude suggests that latewood structural characteristics are more regulated by climate and less influenced by tree age, consistent with studies on some temperate conifers (Cuny and Rathgeber, 2016).

Tree age is an important endogenous factor regulating xylem formation in trees, and its effects are reflected at the molecular level by significant temporal reprogramming of gene expression. Transcriptomic studies in *Larix gmelinii* have shown that with increasing tree age (Li et al., 2017), genes involved in the ethylene signaling pathway (ERF, ACS), calcium signaling (CaM, CML), cell wall expansion and biosynthesis (expansins, cellulose synthases, XTH), as well as transcription factors such as MYB and WRKY, exhibit significant age-dependent expression changes. These genes collectively regulate tracheid growth, cambial cell division activity, cell expansion, and secondary cell wall deposition (Meinzer et al., 2011). From young to middle-aged stages, genes related to xylem development are overall up-regulated, leading to strong cambial activity and high xylem production (Zhang et al., 2024). In contrast, during mature and old stages, numerous growth- and hormone-responsive genes are down-regulated, resulting in reduced rates of xylem formation and cell expansion capacity.

Collectively, these results highlight the significant effects of altitude and tree age class on xylem development and seasonal growth dynamics in *Pinus koraiensis*. Small-age trees show greater sensitivity to temperature limitation at high altitudes but maintain stronger radial growth potential under favorable conditions, whereas large-age trees prioritize stability and stress resistance. These findings improve our understanding of age-related growth strategies in montane conifers and provide data support for forest regeneration, adaptive management, and carbon sink assessment under ongoing climate change. Given the relatively limited sample size in this study, further long-term monitoring with expanded replication is encouraged to verify these patterns.

### 4.3 Limitations

We acknowledge that the entire study is based on observations from a single growing season (2024). The representativeness of meteorological conditions in 2024 relative to long-term averages may remain uncertain. The interannual variability of weather patterns, including fluctuations in temperature, precipitation, and solar radiation, can substantially influence plant growth and physiological responses, and a single-year dataset inevitably lacks the capacity to capture this temporal variability or to determine whether the observed growing conditions were typical of the region’s climatic norms-. As a result, the generalizability of our findings beyond the specific environmental context of the 2024 growing season is constrained, and we caution against extrapolating the conclusions to years with markedly different meteorological conditions. However, we note that numerous studies in agricultural and ecological research have been conducted using data from a single growing season or a single year of field observations, and such studies have been successfully published in peer-reviewed journals. For example, Jones et al. (2025) argued that single-year field experiments can provide valid data when supported by mechanistic understanding and appropriate study design, and that research objectives should determine experiment duration rather than fixed multi-year mandates. Yan and Rajcan (2003) further demonstrated that a single-year, multiple-location trial had sufficient power for identifying genotypes that would perform well or poorly in the next year, with two to four years of data offering only marginally better predictive ability than single-year data. These precedents indicate that while multi-year experiments remain valuable for capturing interannual variability, single-year studies are nonetheless feasible and contribute meaningful insights, particularly when the research addresses specific, timely questions or investigates mechanisms that are well understood. To place our results in a broader temporal context, we have compared our meteorological data with long-term climate records for the study region where available, and we suggest that future work should extend the present experiment over multiple growing seasons to assess the temporal consistency of the observed responses and to evaluate how interannual climate variation modulates treatment effects.

## 5 Conclusion

This study systematically investigated the dynamics of xylem formation in *Pinus koraiensis* across elevation gradients and age classes, clarifying the combined regulatory effects of temperature and tree age on tracheid development and wood structural traits. Our results demonstrate that increasing elevation and associated low temperatures significantly constrain the timing and duration of cambial activity, thereby reducing tracheid production and annual radial growth. Meanwhile, trees at higher elevation allocate more time and resources to cell wall thickening, representing an adaptive strategy to enhance mechanical stability under cold stress. Compared with large-age individuals, small-age trees have a longer effective growth period and higher cell production efficiency, but show greater sensitivity to low-temperature stress at high altitudes due to limited carbon reserves and weaker cold resistance. These findings highlight the age-dependent adaptive strategies of xylem development in response to mountain environments, and improve our understanding of how conifers balance growth, defense, and resource allocation under climate change. This study provides key physiological insights for predicting forest growth dynamics and guiding adaptive silviculture and regeneration management of *Pinus koraiensis* forests in temperate mountainous regions.

## Data Availability

The original contributions presented in the study are included in the article/supplementary material. Further inquiries can be directed to the corresponding author.
